# A Case of Poisoning by Hyoscin Hydrobromid

**Published:** 1904-02

**Authors:** 


### A CASE OF POISONING BY HYOSCIN HYDROBROMID.

F. Krauss, Philadelphia (*N. Y. Medical Journal*, December 12, 1903) relates the case of a patient aet. fifteen years, into each of whose conjunctional sacs 1-120 gr. of this drug was instilled. This was followed by the symptoms of hyoscin poisoning, which fortunately passed off, without treatment, with return of the accommodation to the normal in three days.

